# Definition of Sarcopenia in Chronic Liver Disease

**DOI:** 10.3390/life11040349

**Published:** 2021-04-16

**Authors:** Seong Wan Son, Do Seon Song, U Im Chang, Jin Mo Yang

**Affiliations:** Department of Internal Medicine, St. Vincent’s Hospital, College of Medicine, The Catholic University of Korea, Seoul 06591, Korea; ssw3091@naver.com (S.W.S.); cui70@catholic.ac.kr (U.I.C.); jmyangdr@catholic.ac.kr (J.M.Y.)

**Keywords:** sarcopenia, chronic liver disease, definition, skeletal muscle, muscle strength, cutoff

## Abstract

Sarcopenia, which is characterized by decline in muscle mass, muscle strength, and physical performance, is common in patients with chronic liver disease (CLD) and is associated with poor clinical outcomes. Several consensus definitions for community-dwelling elderly people have been proposed, and these recommend the use of various tools and tests to assess muscle properties and performance. These measurement tools have also been applied in patients with CLD and have been useful for predicting prognosis. However, sarcopenia and its diagnostic criteria specific to patients with CLD have not yet been clearly defined. In addition, fluid retention and body composition should be considered when sarcopenia is assessed in patients with CLD. This review aims to introduce definitions of sarcopenia and diagnostic tools used in patients with CLD.

## 1. Introduction

Sarcopenia is defined as a syndrome in which muscle mass and muscle function (muscle strength and/or physical performance) progressively decrease with age [1,2]. Sarcopenia has been considered a geriatric syndrome that leads to functional limitations and metabolic dysregulation in older people [3]. In addition to age, muscle mass and muscle function are also affected by gender, physical activities, and drugs [4,5,6,7]. However, sarcopenia can occur secondary to a systemic disease, including chronic liver disease (CLD) [1,2].

Sarcopenia is common in cirrhotic patients, occurring in 30%–70% [8]. The cause of sarcopenia in patients with cirrhosis is multifactorial, including decreased nutritional intake, malabsorption, altered metabolism, hormonal changes, hyperammonemia, and increased muscle loss [9]. Sarcopenia is not only associated with a higher rate of complications, such as infections, hepatic encephalopathy, and ascites [10,11,12], but is also an independent predictor of a high mortality rate in cirrhotic patients [13,14]. Therefore, sarcopenia has been recognized as a crucial complication of cirrhosis, and the clinical practice guidelines of the European Association for the Study of the Liver [15] and European Society for Clinical Nutrition and Metabolism [16] recommend screening for malnutrition and sarcopenia in cirrhotic patients. In addition, sarcopenia is associated with metabolic syndrome and advanced fibrosis in noncirrhotic CLD patients [17,18,19].

Sarcopenia has been studied mainly in community-dwelling older individuals, and several consensus definitions have been developed [1,2,20]. However, it is difficult to apply the existing consensus definition to patients with cirrhosis because the measurement of muscle mass in cirrhotic patients might be influenced by hepatic function and water retention, such as ascites or peripheral edema. In addition, there is a lack of consensus on which sarcopenia definition to use in patients with CLD. Therefore, the purpose of this review is to discuss the tools for the assessment of sarcopenia and definitions that can be used in patients with CLD.

## 2. Consensus Definition of Sarcopenia

The European working group on sarcopenia in older people (EWGSOP), and Asian working group for sarcopenia (AWGS) proposed consensus definitions for age-related sarcopenia [1,2,21,22]. In 2010, the EWGSOP defined sarcopenia as occurring when both muscle mass and muscle function (muscle strength or physical performance) declined in older subjects (EWGSOP1) [21]. In the revised definition from the EWGSOP1 in 2018 (EWGSOP2), muscle mass included muscle quality as well as quantity, and low muscle strength was used as the primary parameter for sarcopenia [2]. The AWGS proposed a definition similar to that of the EWGSOP in 2014 and 2019, in which low muscle mass and low muscle strength or low physical performance were required for the diagnosis of sarcopenia [1,22].

These definitions recommended measurement tools: (1) computed tomography (CT), magnetic resonance imaging (MRI), bioelectrical impedance analysis (BIA), or dual energy X-ray absorptiometry (DXA) for muscle mass; (2) handgrip strength (HGS) for muscle strength; and (3) gait speed (GS) or chair stand test for physical performance. While EWGSOP1 did not advise specific cutoff values, EWGSOP2 provided the cutoff values, which were defined by two standard deviations (SDs) below the mean reference values (healthy young European adults) [2]. The AWGS also recommended cutoff values based on studies of sarcopenia in Asian subjects (lower than 2 SD below the mean muscle mass of the young reference group) [1,22], but these cutoff values were different from those in the EWGSOP definition. Even within the same population, the prevalence of sarcopenia can differ according to the definition because of the use of different cutoff values [23,24].

The Foundation for the National Institutes of Health Sarcopenia (FNIH) Project proposed consensus definitions from nine sources of community-dwelling elderly cohorts [25]. This definition recommended sex-specific cutoff values for grip strength and appendicular lean body mass measured by DXA.

The Japanese Society of Hepatology (JSH) established criteria for sarcopenia specific to liver disease patients [26], which define sarcopenia based on a combination of low muscle mass and low HGS. The JSH definition provides cutoff values for the diagnosis of sarcopenia. Most cutoff values for the JSH definition were adopted from AWGS or derived from studies involving Japanese patients.

## 3. Muscle Mass Measurement

### 3.1. Skeletal Muscle Measurement by CT

The measurement of skeletal muscle on cross-sectional CT or MRI images is considered the gold standard for the evaluation of sarcopenia [27]. The skeletal muscle area of a single abdominal cross-sectional image is highly correlated with the total skeletal muscle volume, and the skeletal muscle area at the third lumbar (L3) level is the most highly correlated [28]. Therefore, muscle mass measurements are performed at the L3 level in most sarcopenia studies using CT imaging. The L3 region contains the psoas, paraspinal muscles (erector spinae, quadratus lumborum), and abdominal wall muscles (transversus abdominus, external and internal obliques, rectus abdominus). Skeletal muscles are identified and quantified by Hounsfield unit thresholds of −29 to 150 [29]. Because absolute skeletal muscle volume is strongly correlated with height, the skeletal muscle index (SMI), which is normalized for stature (cm^2^/m^2^), is used in sarcopenia studies [30].

The most commonly used cutoff points for the SMI in sarcopenia studies are 52.4 cm^2^/m^2^ for men and 38.5 cm^2^/m^2^ for women, which were found to be associated with mortality in obese patients with solid tumors [31]. These sex-specific cutoff values have also been widely used in sarcopenia studies in patients with cirrhosis and have been shown to be associated with high mortality [13,32,33,34,35,36,37]. Martin et al. developed another cutoff value derived from cancer patients, which was stratified by body mass index, and sarcopenia based on this cutoff value was associated with increased mortality (L3 SMI ≤ 53 cm^2^/m^2^ for men and ≤41 cm^2^/m^2^ for women with a BMI ≥ 25 kg/m^2^ and L3 SMI ≤ 43 cm^2^/m^2^ for patients with a BMI < 25 kg/m^2^) [38]. These cutoff values were associated with increased wait-list mortality [39], longer posttransplant hospital stay and a higher rate of postoperative bacterial infection but not increased mortality [40]. Carey et al. reported new sex-specific cutoffs (50 cm^2^/m^2^ for men and 39 cm^2^/m^2^ for women) that could be used to identify groups with high waitlist mortality in patients with end-stage liver disease [41]. However, because studies to validate the SMI cutoffs proposed by Carey et al. did not show differences in waitlist mortality between patients with and without sarcopenia [39,42], this cutoff needs to be validated. JSH established SMI cutoff values (42 cm^2^/m^2^ for men and 38 cm^2^/m^2^ for women) for patients with CLD [26]. These cutoffs, which are equivalent to those for BIA, are lower than those established in Western countries. On the other hand, Ebadi et al. defined severe sarcopenia as −1 SD below the sex-specific mean value in young liver transplant donors (50 cm^2^/m^2^ for men and 37 cm^2^/m^2^ for women) for patients with cirrhosis [43]. In this study, sarcopenic patients had shorter survival than patients without sarcopenia, but these cutoffs also need to be validated. The various cutoffs for the SMI that have been used in patients with CLD are summarized in Table 1.

CT scans are commonly used in clinical practice for patients with CLD. The SMI measured by CT scanning is reproducible, accurate, and objective [13]. CT scanning can also assess myosteatosis (muscle quality), which is characterized by the pathologic accumulation of fat in skeletal muscle [48]. In addition, three-dimensional quantitative color CT can measure the atrophied muscle volume and normal muscle volume separately [49,50]. However, specific software is needed to measure the cross-sectional area of skeletal muscle. In addition, fluid retention could lead to the overestimation of muscle mass [51]. Furthermore, there are no cutoff points for the diagnosis of sarcopenia, usually defined as −2 SD below the reference, due to the lack of a reference value for the SMI in young healthy adults.

### 3.2. Psoas Muscle Measurement by CT

Several psoas muscle measurement methods, including psoas muscle thickness (PMT), psoas muscle area (PMA) and psoas muscle index (PMI), have been used to predict outcomes in patients with CLD. Sarcopenia defined by psoas muscle measurement has been investigated as an important prognostic factor for waitlist mortality [52,53] and post-LT mortality [54,55,56]. The single muscle approach, which uses the psoas muscle to diagnose clinically relevant skeletal muscle depletion, is a simple and convenient method compared to the SMI because specific software is not needed. The muscle mass measured using psoas muscle was found to be well correlated with the SMI in patients with cirrhosis [33,57]. However, the sarcopenia diagnosis methods using the psoas muscle are heterogeneous (Table 2). First, psoas muscle mass can be measured by PMA, which is determined by the sum of the areas of the right and left psoas muscles, or by transversal PMT (TPMT), which is determined as the largest diameter perpendicular to the longest diameter. Second, normalization is not uniform. Among the studies using PMA, some studies used the PMA [54,55,58], and other studies used the PMI, in which PMA is normalized by the squared height [56,57,59,60,61,62]. In studies using the TPMT, the TPMT is normalized by height [14,33,52,53,63]. Third, the psoas muscle measurement level is heterogeneous. Some researchers measured the psoas muscle at the umbilicus level [14,33,52,53,56,63], whereas most SMIs were measured at the L3–4 level. The umbilicus level can be easily identified on CT scans, preventing errors owing to the inability to identify the precise lumbar level due to sacralization of the L5 vertebra, lumbar wedge fractures or pronounced lordosis [52]. However, the umbilical level can be changed by the presence of ascites.

It is controversial as to whether the SMI or psoas muscle assessment is more useful for assessing sarcopenia in patients with CLD. Golse et al. reported that the PMA is more predictive of post-LT one-year survival than the L3-SMI [54]. However, Wells showed that the abdominal skeletal muscle area was more strongly correlated with total-body protein, representing whole-body protein stores, than the PMA [51]. In addition, the PMI had poor concordance with the SMI for the diagnosis of sarcopenia, and the PMI was not an independent factor for waitlist mortality in men. Therefore, further research is needed on the role of psoas muscle assessment in the evaluation of sarcopenia in patients with CLD.

### 3.3. Dual Energy X-ray Absorptiometry (DXA)

DXA measures the relative attenuation of two different energy X-rays by human tissue. It can measure three body components: fat, bone minerals and lean tissue [64]. Appendicular skeletal muscle (ASM) accounts for >75% of skeletal muscle, and its reduction leads to weakness, disability, impaired quality of life, and increased mortality [2,65]. Therefore, ASM measurement has been used to evaluate sarcopenia. ASM is the sum of the muscle mass of the four limbs. As muscle mass is correlated with body size, sarcopenia indices, in which ASM is normalized by the squared height (ASM index, ASMI), weight or body mass index (ASM_BMI), are used for the diagnosis of sarcopenia (Table 3).

The ASMI was found to be well correlated with the SMI measured by CT in patients with cirrhosis [45,66]. The cutoff values for the diagnosis of sarcopenia in elderly people were usually determined to be below 1 SD or 2 SD of the value in healthy young adults [30,67]. Baumgartner et al. proposed cutoff values for the ASMI, determined as below 2 SD of the value in healthy young adults: men < 7.26 kg/m^2^ and women < 5.45 kg/m^2^ [30]. This sex-specific cutoff value was adopted in the EWGSOP1 definition [21] and has been used in patients with CLD [45,68]. Lindqvist et al. [66] used the sex-specific cutoff value, which was determined as 1 SD below the mean in the young population by Coin et al. [67]. The definitions of low muscle mass using the ASMI in recent revised consensus definitions are as follows: men < 7.0 kg/m^2^ and women < 5.4 kg/m^2^ in the AWGS definition [1,22] and men < 7.0 kg/m^2^ and women < 5.5 kg/m^2^ in the EWGSOP2 definition [2]. These revised criteria were not validated in patients with CLD.

The ASMI is positively correlated with BMI, visceral fat, and insulin resistance, while weight-adjusted or BMI-adjusted sarcopenia indices are negatively correlated in obese subjects [17,69]. This means that those subjects with high BMI are less likely to be classified as having sarcopenia. Therefore, weight-adjusted or BMI-adjusted sarcopenia indices are more appropriate in patients with nonalcoholic fatty liver disease (NAFLD), which is associated with obesity [18,70,71]. The FNIH definition proposed sex-specific cutoff values of the sarcopenia index, in which the ASM is divided by BMI (men < 0.789 and women < 0.521) [25]. This cutoff value has mainly been used in research on NAFLD [18,71]. In addition, some researchers defined the lowest quintile of the ASM_BMI as sarcopenia [19], or defined sarcopenia using upper limb muscle mass [68,72].

Studies on sarcopenia using DXA in patients with CLD have used the muscle depletion criteria of the consensus definition for community-dwelling elderly individuals, and these cutoff values were useful for predicting the outcome in CLD patients [45,68,73,74]. However, the most important limitation of DXA is that it cannot differentiate muscle from water, and water retention can lead to the overestimation of skeletal muscle in patients with cirrhosis [45,66]. A recent study reported that the ASMI measured by DXA is not influenced by ascites or edema and is useful for the prediction of mortality in patients with cirrhosis [74]. Therefore, future studies using DXA are needed to establish the definition of sarcopenia applicable to patients with CLD without the effects of ascites or edema.

**Table 3:** Studies measuring appendicular skeletal muscle mass using dual-energy X-ray absorptiometry and bioelectrical impedance in patients with chronic liver disease.

Modality	Author (Year)	Country	Number of Subjects	Study Population	Measurement Method	Cutoff for Sarcopenia	Method of Determining Cutoff
DXA	Giusto (2015) [45]	Italy	139	LT candidates	ASMI	7.26 kg/m^2^ for men 5.45 kg/m^2^ for women	Baumgartner [30] (2 SD below the sex-specific mean value)
	Bering (2018) [73]	Brazil	104	Chronic hepatitis C	ASMI	7.26 kg/m^2^ for men 5.45 kg/m^2^ for women	EWGSOP1
	Belarmino (2018) [74]	Brazil	144	Male cirrhotic patients with ascites	ASMI	7.26 kg/m^2^	EWGSOP1
	Alferink (2019) [75]	Netherland	4609	Participants from The Rotterdam Study	ASMI	7.25 kg/m^2^ for men 5.67 kg/m^2^ for women	EWGSOP1
	Sinclair (2019) [68]	Australia	420	Male advanced cirrhosis patients	(1) ASMI (2) Upper limb muscle	(1) 7.26 kg/m^2^ (2) 1.6 kg/m^2^	(1) Baumgartner [30] (2) Mortality-based
	Lindqvist (2019) [66]	Sweden	53	LT recipients	ASMI	7.59 kg/m^2^ for men 5.47 kg/m^2^ for women	Coin (1 SD below the mean in the young population) [67]
	Lee (2016) [18]	Korea	2761	NAFLD subjects defined by NAFLD liver fat score	ASM/BMI	0.789 for men 0.521 for women	FNIH
	Han (2020) [70]	Korea	7191	NAFLD subjects defined by NAFLD prediction models	ASM/BMI	0.882 for men 0.582 for women	Lowest quintile
	Golabi (2020) [71]	USA	4611	NAFLD subjects defined by fatty liver index	ASM/BMI	0.789 for men 0.521 for women	FNIH
	Santos (2021) [72]	Brazil	210	Cirrhotic patients	Upper limb muscle/height^2^	2.104 for men 1.506 for women	ROC curve for mortality
BIA	Nishikawa (2017) [76]	Japan	382	Liver cirrhosis	ASMI	7.0 kg/m^2^ for men 5.7 kg/m^2^ for women	AWGS and JSH definition
	Hanai (2017) [77]	Japan	120	Liver cirrhosis	ASMI	7.0 kg/m^2^ for men 5.7 kg/m^2^ for women	AWGS and JSH definition
	Hayashi (2018) [78]	Japan	112	Chronic liver disease	ASMI	7.0 kg/m^2^ for men 5.7 kg/m^2^ for women	AWGS and JSH definition
	Kim (2020) [79]	Korea	2168	Chronic liver disease who underwent transient elastography	ASM/BMI	0.789 for men 0.521 for women	FNIH
	Seo (2020) [80]	Korea	4210	Men with Type 2 DM	(ASM/body weight) × 100 ASM/BMI	29.0% for men 22.9% for women 0.789 for men 0.521 for women	2 SD below the sex-specific means for in healthy young adults FNIH
	Petta (2017) [81]	Italy	225	NAFLD	(ASM/body weight) × 100	37% for men 28% for women	Janssen

Abbreviations: ASM, appendicular skeletal muscle; ASMI, appendicular skeletal muscle index; AWGS, Asian Working Group for Sarcopenia; BMI, body mass index; DM, diabetes mellitus; EWGSOP, European Working Group on Sarcopenia in Older People; FNIH, Foundation for the National Institutes of Health; JSH, Japan Society of Hepatology; LT, liver transplantation; NAFLD, nonalcoholic fatty liver disease; ROC, receiver operating characteristics; SD, standard deviation.

### 3.4. Bioelectrical Impedance Analysis (BIA)

BIA is a technique in which the body composition of a biological subject is analyzed by measuring its electrical impedance [82]. Initially, BIA used a single frequency, but recent studies showed that multifrequency BIA had better accuracy for the measurement of body composition than single-frequency BIA [83,84], and showed good correlation with DXA [85,86] and the SMI obtained with CT [26]. Similar to DXA, BIA uses the ASM to define low muscle mass, and the ASM is normalized by height, body weight, or BMI (Table 3).

Interestingly, most studies using BIA in patients with CLD were performed in Asian regions. Studies conducted in Japan used the sex-specific cutoff values for the ASMI recommended by the JSH definition (men < 7.0 kg/m^2^ and women < 5.4 kg/m^2^) [26], which is the same as the AWGS definition [22]. Studies in noncirrhotic CLD patients have used sarcopenic indices in which the ASM is normalized to BMI or body weight [79,80,81]. The cutoff value for the ASM/BMI was adopted from FNIH [20], and those for the ASM/weight were determined by 2 SD below the mean reference value in the healthy young population.

The main limitation of BIA in patients with CLD is that the measurement of muscle by BIA can be influenced by water retention [87,88]. Although the ASM measured by BIA had a good correlation with skeletal muscle mass measured by CT [26,89], the measurement of muscle by BIA can be overestimated in patients with ascites. Therefore, most of the studies on sarcopenia using BIA were performed in patients with noncirrhotic CLD or cirrhotic patients without massive ascites.

Recently, the phase angle (PA) obtained from BIA has been proposed as a nutritional status marker in patients with CLD [90,91]. The PA reflects cell membrane integrity and vitality and represents the quantity and quality of soft tissues [92]. The PA was found to be correlated with skeletal muscle mass [93], and PA ≤ 4.9° was an independent factor that was predictive of mortality and hepatic encephalopathy in patients with cirrhosis [74,91,94]. Another study also showed that a PA ≤ 5.05° was a good predictor of sarcopenia in cirrhotic patients [90]. However, research on the PA in patients with CLD is still lacking, and further studies are needed to determine the role of the PA in patients with CLD.

## 4. Muscle Strength and Physical Performance

### 4.1. Handgrip Strength (HGS)

HGS is a simple and inexpensive method of assessment and is the most widely used tool for measuring muscle strength. Low HGS is associated with poor outcomes, such as increased mortality and decreased cognitive function and mobility in the elderly population [95,96]. Consensus definitions recommended the measurement of HGS for the evaluation of muscle strength, and cutoff values were proposed [1,2,21,25]. The EWGSOP1 definition introduced two sex-specific cutoff values for HGS: (1) <30 kg for men and <20 kg for women, as proposed by Laurentani [97], and (2) ≤29 kg if BMI was ≤24 kg/m^2^, ≤30 kg if BMI was 24.1 to 28 kg/m^2^, ≤32 kg if BMI was >28 kg/m^2^ for men and ≤17 kg if BMI was ≤23 kg/m^2^, ≤17.3 kg if BMI was 23.1 to 26 kg/m^2^, ≤18 kg if BMI was 26.1 to 29 kg/m^2^, and ≤21 kg if BMI was >29 kg/m^2^ for women, as proposed by Fried [98]. In 2018, the EWGSOP2 proposed a new cutoff value adopted from the meta-analysis by Dodds et al. (<27 kg for men and <16 kg for women) [99]. The AWGS definition in 2014 also recommended a sex-specific cutoff value defined by the lowest quintile in two studies: <26 kg for men and <18 kg for women [22]. This cutoff value was revised in the 2019 AWGS definition, defined by the lowest quintile in eight studies: <28 kg for men and <18 kg for women [1]. JSH recommended the same cutoff value as the AWGS 2014 [26].

Most studies in CLD patients have used cutoff values from the consensus definition for community-dwelling elderly populations (EWGSOP1 and AWGS 2014) (Table 4). Some researchers found new cutoff values that could predict outcomes in their study population by ROC curve analysis [100,101,102]. Although the diagnosis of sarcopenia using those cutoffs has been found to be a strong predictor of poor outcomes in patients with CLD [100,101,102,103], further research is needed to determine which cutoff values are the most useful in patients with CLD, and validation of the cutoff values in the revised consensus definitions (EWGSOP2 and AWGS 2019) is needed. Consideration should be given to the fact that the cutoffs may vary depending on the type of dynamometer and the measurement protocol [104].

### 4.2. Chair Stand Test

The chair stand test is used to assess lower extremity strength. The chair stand test measures the time needed for a patient to rise five times from a seated position without using the arms or the number of chair stands completed in 30 s. AWGS 2019 and EWGSOP2 recommend the chair stand test as a parameter for physical performance and muscle strength, respectively. The cutoff values in these consensus definitions were 12 s in AWGS and 15 s in EWGSOP2 [1,2].

The chair stand test in patients with CLD has been used as a parameter for frailty rather than sarcopenia [109,110,111]. The chair stand test was found to be an independent factor predictive of poor quality of life and waitlist mortality in patients with end-stage liver disease [110,112,113]. However, research on the usefulness of and appropriate cutoff values for the chair stand test in patients with CLD is still lacking.

### 4.3. Gait Speed

GS is the most frequently used tool for the assessment of physical performance. GS was found to be associated with the development of cirrhotic complications, waitlist mortality, and post-LT complications in patients with cirrhosis [114,115,116,117]. In the consensus definition for community-dwelling elderly people, the recommended cutoff value for the diagnosis of low physical performance was 0.8 m/s regardless of sex [1,2]. Some studies used 1.0 m/s as the cutoff, but most studies on GS in patients with CLD have used 0.8 m/s as the cutoff value (Table 5).

## 5. Considerations for the Diagnosis of Sarcopenia and Future Prospects in Patients with Chronic Liver Disease

The definition of sarcopenia is progressive decline in muscle mass and muscle function. Therefore, consensus definitions for elderly people define sarcopenia based on the presence of both low muscle quantity and low muscle strength and/or low physical performance [1,2]. However, studies on whether it is appropriate to apply these definitions to patients with CLD are lacking. Only a few studies have used a combination of muscle mass and muscle function to diagnose sarcopenia in patients with CLD [73,74,75,77,78], and more studies have been performed on decreased muscle mass than on decreased muscle strength or physical performance. In patients with CLD, further studies are needed to determine which definition is the most useful for predicting a poor outcome among decreased muscle mass, decreased muscle function and both.

There are many diagnostic tools and tests available for the assessment of sarcopenia, and each test has variations. For example, muscle mass measurement using a single cross-sectional image obtained from CT includes the measurement of the skeletal muscle area and psoas muscle mass, and the psoas muscle mass measurement includes the psoas muscle area and transverse thickness. The muscle measurement levels include L3–4 or the umbilicus, and the measured muscle mass is normalized by the height or the square of the height. The HGS test also relies on various dynamometer types and measurement protocols. These heterogeneous protocols make it difficult to derive a unified definition in patients with CLD. Therefore, future studies are needed to find appropriate protocols that can accurately measure muscle mass or muscle function and predict prognosis in patients with CLD.

Due to the lack of cutoff values specific to patients with CLD, most sarcopenia studies in patients with CLD have been performed using preexisting cutoffs, such as those defined in patients with cancer [31] or those included in consensus definitions for community-dwelling elderly people [1,2,20,21,22]. These cutoffs were also effective in predicting poor outcomes even when used in CLD patients. However, in patients with CLD, fluid retention and body composition should be considered. Since the measurement of muscle mass, especially with DXA or BIA, can be influenced by overhydration in patients with cirrhosis, further studies are needed on whether it is appropriate to apply consensus definitions to CLD patients. Therefore, it is necessary to find and validate CLD specific cutoff values of skeletal muscle mass and muscle function, which are more useful for predicting poor outcomes, such as mortality or the development of complications, in CLD patients. Although JSH proposed a sarcopenia definition for patients with CLD, most cutoff values in the JSH definition were adopted from AWGS or derived from studies with Japanese patients [26]. The JSH definition needs to be validated in CLD patients of various ethnicities. The SMI cutoff value, defined from obese patients with solid tumors using CT, has shown usefulness in prognosis prediction, mainly in advanced cirrhosis patients [13,36,44,45]. A cutoff value for skeletal muscle mass using CT derived from patients with end-stage liver disease was recently proposed [41], which also needs to be further validated, and comparative studies with preexisting SMI cutoff are needed.

## 6. Conclusions

Sarcopenia is related to a poor prognosis and the occurrence of cirrhotic complications. Many studies on sarcopenia have been conducted in patients with CLD due to its clinical implications, but an appropriate definition of sarcopenia and optimal protocols have still not been established. In addition, specific cutoff values for measuring muscle mass and muscle function in CLD patients need to be identified and validated. We hope that a standardized working definition for patients with CLD can be identified that can be used to accurately predict adverse outcomes in future studies.

## Figures and Tables

**Table 1:** Studies measuring skeletal muscle area using computed tomography in patients with chronic liver disease.

Author (Year)	Country	Number of Subjects	Study Population	Level of Measurement	Cutoff for Sarcopenia
Montano-Loza (2012) [13]	Canada	112	LT candidates	L3	52.4 cm^2^/m^2^ for men 38.5 cm^2^/m^2^ for women
Tandon (2012) [44]	Canada	142	LT candidates	L3	52.4 cm^2^/m^2^ for men 38.5 cm^2^/m^2^ for women
DiMartini (2013) [37]	U.S.A.	338	LT candidates	L3–4	52.4 cm^2^/m^2^ for men 38.5 cm^2^/m^2^ for women
Giusto (2015) [45]	Italy	59	LT candidates	L3	52.4 cm^2^/m^2^ for men 38.5 cm^2^/m^2^ for women
Hanai (2015) [46]	Japan	130	Cirrhosis	L3	52.4 cm^2^/m^2^ for men 38.5 cm^2^/m^2^ for women
Sinclair (2016) [34]	Australia	145	Male LT candidates	L4	52.4 cm^2^/m^2^ for men 38.5 cm^2^/m^2^ for women
Gu (2018) [33]	Korea	653	Cirrhosis	L3	52.4 cm^2^/m^2^ for men 38.5 cm^2^/m^2^ for women
Paternostro (2019) [36]	Austria	109	Cirrhosis	L3	52.4 cm^2^/m^2^ for men 38.5 cm^2^/m^2^ for women
Acosta (2019) [32]	U.S.A.	119	Patients who underwent LT for HCC	L3	52.4 cm^2^/m^2^ for men 38.5 cm^2^/m^2^ for women
Kumar (2020) [35]	India	115	LT recipients	L3	52.4 cm^2^/m^2^ for men 38.5 cm^2^/m^2^ for women
Carey (2017) [41]	North America	396	LT candidates	L3	50 cm^2^/m^2^ for men 39 cm^2^/m^2^ for women
Ebadi (2018) [47]	North America	353	LT candidates	L3	50 cm^2^/m^2^ for men 39 cm^2^/m^2^ for women
Kappus (2020) [42]	U.K.	355	LT candidates	L3	50 cm^2^/m^2^ for men 39 cm^2^/m^2^ for women
Montano-Loza (2014) [40]	Canada	248	LT candidates	L3	Male 53 cm^2^/m^2^ for BMI ≥ 25 kg/m^2^ 43 cm^2^/m^2^ for BMI < 25 kg/m^2^ Female 41 cm^2^/m^2^
van Vugt (2018) [39]	Netherland	585	Patients with cirrhosis listed for LT	L3	1. Martin et al. [38] Male 53 cm^2^/m^2^ for BMI ≥ 25 kg/m^2^ 43 cm^2^/m^2^ for BMI < 25 kg/m^2^ Female 41 cm^2^/m^2^ 2. Carey et al. [41] 50 cm^2^/m^2^ for men 39 cm^2^/m^2^ for women
Nishikawa (2016) [26]	Japan	149	Chronic liver disease	L3	42 cm^2^/m^2^ for men 38 cm^2^/m^2^ for women
Ebadi (2020) [43]	Canada	603	Cirrhosis	L3	50 cm^2^/m^2^ for men 37 cm^2^/m^2^ for women

Abbreviations: HCC, hepatocellular carcinoma; LT, liver transplantation.

**Table 2:** Studies measuring psoas muscle mass using computed tomography in patients with chronic liver disease.

Author (Year)	Country	Number of Subjects	Study Population	Measurement Method	Level of Measure	Cutoff for Sarcopenia	Method of Determining Cutoff
Englesbe (2010) [55]	USA	163	Cirrhosis patients undergoing LT	PMA	L4	NR	Lowest quartiles
Krell (2013) [58]	USA	207	Adult patients undergoing LT	PMA	L4	NR	Lowest tertile
Golse (2017) [54]	USA	256	Cirrhosis patients undergoing LT	PMA	L3-4	Male: 1561 mm^2^ Female: 1464 mm^2^	ROC curve for 1-year mortality
Hamaguchi (2014) [56]	Japan	200	Adult patients undergoing LT	PMA/height^2^	Umbilicus	Male: 6.868 cm^2^/m^2^ Female: 4.117 cm^2^/m^2^	ROC curve for mortality
Tsien (2014) [62]	USA	53	Adult patients undergoing LT	PMA/height^2^	L4	Age <50 years Male: 12.3 cm^2^/m^2^, Female: 10.5 cm^2^/m^2^ Age >50 years Male: 10.1 cm^2^/m^2^, Female: 10.3 cm^2^/m^2^	Age- and sex-specific 5th percentile values
Kalafateli (2017) [60]	UK	232	LT recipients	PMA/height^2^	L3	Male: 340 mm^2^/m^2^ Female: 264 mm^2^/m^2^	Lowest sex-stratified quartiles
Ebadi (2018) [57]	North America	353	Patients with cirrhosis on waiting list	PMA/height^2^	L3	Male: 5.1 cm^2^/m^2^ Female: 4.3 cm^2^/m^2^	ROC curve for waitlist mortality
Hou (2020) [59]	China	251	Cirrhotic patients	PMA/height^2^	L3	Male: 3.5 cm^2^/m^2^ Female: 2.6 cm^2^/m^2^	ROC curve for 3-year mortality
Ohara (2020) [61]	Japan	318	Chronic liver disease	PMA/height^2^	L3	(1) Male: 330 mm^2^/m^2^ Female: 169 mm^2^/m^2^ (2) Male: 374 mm^2^/m^2^ Female: 229 mm^2^/m^2^	(1) Less than 2 SD below the mean of normal control (2) The bottom 5% or normal control
Durand (2014) [52]	France	562	Patients with cirrhosis on waiting list	TPMT/height	Umbilicus	16.8 mm/m	Youden index for waitlist mortality
Kim (2014) [14]	Korea	65	Decompensated cirrhosis	TPMT/height	L3–4 or umbilicus	14 mm/m	ROC curve for 1-year mortality
Gu (2018) [33]	Korea	653	Cirrhotic patients	TPMT/height	Umbilicus	16.8 mm/m	Previous reference
Huguet (2018) [53]	France	173	Cirrhotic patients on waiting list	TPMT/height	Umbilicus	15.2 mm/m	ROC curve for mortality
Praktiknjo (2019) [63]	Europe	186	Patients with decompensated cirrhosis who underwent the TIPS	TPMT/height	Umbilicus	Male: 17.8 mm/m Female: 14.0 mm/m	ROC curve for 1-year mortality

Abbreviations: LT, liver transplantation; PMA, psoas muscle area; ROC, receiver operating characteristics; SD, standard deviation; TIPS, trans-jugular intrahepatic portosystemic shunt; TPMT, transversal psoas muscle thickness.

**Table 4:** Studies measuring handgrip strength in patients with chronic liver disease.

Author (Year)	Country	Number of Subjects	Study Population	Dynamometer	HGS Analysis	Hand	Position	Repetitions	Cutoff for Sarcopenia	Method of Determining Cutoff
Lai (2014) [105]	USA	294	LT candidate	NR	Highest value	Dominant	NR	3	Cutoff according to sex and BMI	EWGSOP1 (Fried)
Augusti (2016) [100]	Brazil	54	Cirrhosis	Saehan dynamometer	Highest value	Nondominant	Sitting	3	20.5 kg	ROC curve analysis for discriminating hepatic encephalopathy
Hiraoka (2016) [106]	Japan	807	Chronic liver disease	TL110 (Toei Light Co.)	Mean of the best value of both hand	Both	Standing	A few measurements	26 kg for men 18 kg for women	AWGS definition (2014)
Wang (2016) [107]	USA	292	Adults listed for LT	Jamar hydraulic dynamometer	Mean value	Dominant	NR	3	Cutoff according to sex and BMI	EWGSOP1 (Fried)
Belarmino (2017) [74]	Brazil	144	Male patients with cirrhosis	Digital dynamometer (Charder Co. Ltd.)	Higher value	Nondominant	NR	3	30 kg for men	EWGSOP1 (Laurentani)
Daphnee (2017) [101]	India	180	LT candidate	Jamar hydraulic dynamometer	Mean value	Dominant	Sitting	3	19.5 kg	ROC curve analysis for discriminating survivor
Harimoto (2017) [108]	Japan	366	Patients who underwent LDLT	Digital grip strength dynamometer	Higher value	NR	NR	2	26 kg for men 18 kg for women	AWGS definition (2014)
Alferink (2019) [75]	Netherland	4609	Participants from The Rotterdam Study	Hydraulic dynamometer (Fabrication Enterprises Inc.)	Highest value	Nondominant	NR	3	Cutoff according to sex and BMI	EWGSOP1 (Fried)
Hanai (2019) [102]	Japan	563	Cirrhosis	T.K.K5101 GRIP-D (Takei Scientific Instruments)	Mean of the highest value of both hands	Both	Standing	NR	30 kg for men 20 kg for women	ROC curve analysis for discriminating survivor
Sinclair (2019) [103]	Australia	145	Men referred for LT	Jamar hydraulic dynamometer	Mean value	Nondominant	Sitting	3	30 kg for men	EWGSOP1 (Laurentani)
Traub (2020) [24]	Austria	114	Cirrhosis	Jamar hydraulic dynamometer	NR	NR	NR	NR	(1) EWGSOP1 30 kg for men 20 kg for women (2) EWGSOP2 27 kg for men 16 kg for women	EWGSOP1 (Laurentani) and EWGSOP2 (Dodds)

Abbreviations: AWGS, Asian Working Group for Sarcopenia; BMI, body mass index; DM, diabetes mellitus; EWGSOP, European Working Group on Sarcopenia in Older People; LDLT, living donor liver transplantation; LT, liver transplantation; NR, not reported; ROC, receiver operating characteristics.

**Table 5:** Studies measuring gait speed in patients with chronic liver disease.

Author (Year)	Country	Number of Subjects	Study Population	Distance	GS Analysis	Repetitions	Cutoff for Sarcopenia	Method of Determining Cutoff
Harimoto (2017) [108]	Japan	366	Patients who underwent LDLT	10 m	Higher value	2	0.8 m/s	AWGS
Alferink (2019) [75]	Netherland	4609	Participants from The Rotterdam Study	5.79 m	NR	NR	1. Men Height ≤ 173 cm: 0.65 m/s Height > 173 cm: 0.76 m/s 2. Women Height ≤ 159 cm: 0.65 m/s Height > 159 cm: 0.76 m/s	EWGSOP1 (Fired)
Nishikawa (2020) [118]	Japan	341	Chronic liver disease	6 m	Average value	2	1.0 m/s	Japanese version of the Cardiovascular Health Study criteria
Nishikawa (2020) [119]	Japan	356	Chronic liver disease	6 m	Average value	2	0.8 m/s	EWGSOP and AWGS
Deng (2020) [116]	China	113	Cirrhosis	5 m	Average value	3	0.8 m/s	EWGSOP2
Soto (2021) [117]	Chile	126	Cirrhosis	4 m	NR	NR	0.8 m/s	EWGSOP and AWGS

Abbreviations: AWGS, Asian Working Group for Sarcopenia; EWGSOP, European Working Group on Sarcopenia in Older People; GS, gait speed; LDLT, living donor liver transplantation; NR, not reported.

## Data Availability

No new data were created or analyzed in this study. Data sharing is not applicable to this article.
